# Time course of hemispheric cerebral volume after decompressive craniectomy in malignant middle cerebral artery stroke

**DOI:** 10.1055/s-0043-1764415

**Published:** 2023-05-09

**Authors:** Thiago Pereira Rodrigues, Mariana Athaniel Silva Rodrigues, Leonardo Favi Bocca, Feres Eduardo Chaddad Neto, Sergio Cavalheiro, Edson Amaro Júnior, Gisele Sampaio Silva, Italo Capraro Suriano, Ricardo Silva Centeno

**Affiliations:** 1 Universidade Federal de São Paulo, Departamento de Neurologia e Neurocirurgia, São Paulo SP, Brazil.; 2 Hospital Israelita Albert Einstein, Departamento de Radiologia, São Paulo SP, Brazil.; 3 Hospital Israelita Albert Einstein, Organização de Pesquisa Acadêmica, São Paulo SP, Brazil.

**Keywords:** Decompressive Craniectomy, Infarction, Middle Cerebral Artery, Intracranial Pressure, Craniectomia Descompressiva, Infarto de Artéria Cerebral Média, Pressão Intracraniana

## Abstract

**Background** Brain edema is the leading cause of death in patients with malignant middle cerebral artery (MCA) infarction. Midline shift (MLS) has been used as a monohemispheric brain edema marker in several studies; however, it does not precisely measure brain edema. It is now possible to directly measure hemisphere brain volume. Knowledge about the time course of brain edema after malignant middle cerebral artery infarction may contribute to the condition's management.

**Objective** Therefore, our goal was to evaluate the course of brain edema in patients with malignant MCA infarction treated with decompressive craniectomy (DC) using hemispheric volumetric measurements.

**Methods** Patients were selected consecutively from a single tertiary hospital between 2013 and 2019. All patients were diagnosed with malignant middle cerebral artery infarction and underwent a decompressive craniectomy (DC) to treat the ischemic event. All computed tomography (CT) exams performed during the clinical care of these patients were analyzed, and the whole ischemic hemisphere volume was calculated for each CT scan.

**Results** We analyzed 43 patients (197 CT exams). Patients' mean age at DC was 51.72 [range: 42–68] years. The mean time between the ischemic ictus and DC was 41.88 (range: 6–77) hours. The mean time between the ischemic event and the peak of hemisphere volume was 168.84 (95% confidence interval [142.08, 195.59]) hours.

**Conclusion** In conclusion, the peak of cerebral edema in malignant MCA infarction after DC occurred on the 7th day (168.84 h) after stroke symptoms onset. Further studies evaluating therapies for brain edema even after DC should be investigated.

## Resumo

**Antecedentes** O edema cerebral é a principal causa de morte em pacientes com infarto maligno de artéria cerebral média. O desvio da linha média tem sido utilizado como marcador de edema cerebral mono-hemisférico em alguns estudos; porém, ele não mede de forma precisa o edema cerebral. Atualmente é possível mensurar diretamente o volume do hemisfério cerebral. O conhecimento sobre a evolução temporal do edema cerebral após infartos malignos da artéria cerebral média pode contribuir para o cuidado clínico desta condição.

**Objetivo** Nosso objetivo é avaliar o edema hemisférico ao longo do tempo, em pacientes com infarto maligno da artéria cerebral média, tratados com craniectomia descompressiva.

**Métodos** Os pacientes foram selecionados de forma consecutiva, em um hospital terciário, entre 2013 e 2019. Todos os pacientes apresentavam diagnóstico de infarto maligno de artéria cerebral média e foram submetidos a craniectomia descompressiva. Todas as tomografias computadorizadas de crânio destes pacientes foram analizadas, e o volume do hemisfério cerebral infartado foi mensurado.

**Resultados** Analisamos 43 pacientes (197 tomografias de crânio). A idade média dos pacientes na craniectomia descompressiva foi 51,72 (42–68) anos. O tempo médio entre o ictus e a craniectomia descompressiva foi 41,88 (6–77) horas. O tempo médio entre o ictus e o pico do volume hemisférico foi 168,84 (142,08–195,59) horas.

**Conclusão** O pico do volume cerebral em pacientes com infarto maligno de artéria cerebral média submetidos a craniectomia descompressiva ocorreu no 7o dia (168,84 horas) após o infarto. Mais estudos avaliando terapêuticas direcionadas ao edema cerebral seriam úteis neste contexto.

## INTRODUCTION

Up to 10% of all stroke patients suffer from complete middle cerebral artery (MCA) infarction 1 ; the leading cause of death is brain edema. 2 Swelling of the brain after ischemic insult has been described in numerous studies. 1 3 4 In an autopsy study, Shaw et al. 5 demonstrated that after an infarction in MCA territory, the midline shift (MLS) reaches its peak at day four. Silver et al. 4 found that the peak risk for death secondary to transtentorial herniation after a supratentorial infarction occurred 3 to 5 days (72–120 h) following the ischemic event. More recently, according to computed tomography (CT) exams, Dohmen et al. 3 showed that in 9 patients with a malignant course of an MCA infarction, MLS peaked after 54.7 h.

Midline shift has been used as a marker of monohemispheric brain edema in several studies. 6 7 However, it does not measure brain edema with the required volumetric quantitative precision. Instead, MLS more likely represents subfalcine herniation, 8 which occurs in advanced cases of monohemispheric brain edema. At the onset of brain edema in the cerebral hemisphere, compensatory mechanisms, including a reduction in hemispheric cerebrospinal fluid and blood volumes, occur before the establishment of MLS. 9

Because of such compensatory mechanisms, intracranial pressure (ICP) monitoring in patients with malignant MCA infarction before decompressive craniectomy (DC) has limited use since patients with severe brain stem compression, and even pupillary asymmetry, can present normal ICP values. 10

Therefore, in patients with malignant MCA infarction, cerebral edema's time course determines the therapeutic option and their timing. In this way, comprehensive knowledge regarding the time course of brain edema after DC could contribute to such patients' management.

Regarding this matter, cerebral whole-hemisphere volumetry, and its variation according to time from symptoms onset is an important factor to be considered. There are several free non-commercial tools used in neuroscience in order to measure brain volumes, including: FreeSurfer, volBrain and 3D-Slicer. Among these, 3D-Slicer is the unique option that allows CT analysis (the others are based on magnetic resonance imaging [MRI]). 11

Studying patients with DC after malignant MCA infarction offers a unique opportunity to observe the time course of brain swelling after large ischemic strokes in patients that do not have a rigid covering of the brain. Therefore, our goal was to evaluate the course of brain edema in patients with malignant MCA infarction treated with decompressive craniectomy (DC) using hemispheric volumetric evaluations.

## METHODS

### Patients' selection

This retrospective study was approved by an ethics committee and was performed following the declaration of Helsinki. During hospital admission, signed consent was obtained from patients or relatives, and all data were anonymized at the source. We selected consecutive patients from a single tertiary hospital between 2013 and 2019. All patients had been diagnosed with a malignant MCA infarction and were submitted to DC to treat the ischemic event. Patients with an intracerebral hematoma, either initially or after hemorrhagic transformation, were excluded. Patients with prolonged time between symptoms onset and DC (> 96 hours) were excluded as well, since this prolonged ischemic time in a hemisphere cover by bone could prevent hemisphere to expand.

Diagnosis of malignant MCA infarction was made through a comprehensive analysis of multiple factors, including infarction size, National Institutes of Health stroke scale (NIHSS) score, radiological signs of mass effect, age, and past medical history. Subsequently, a multidisciplinary team that included a neurologist, a neurosurgeon, and a critical care physician decided whether to apply DC or not. Under this diagnosis (malignant MCA infarction) we included patients with a large infarction in the vascular territory of the MCA, including or not impairment of other vascular territories (anterior cerebral artery or posterior cerebral artery).

Clinical and radiological data were collected from the electronic medical records and included: age, sex, side of infarction, time from onset of symptoms until DC, NIHSS score, pupillary status before DC, hemispheric volume time course variation, infarction vascular territory, past medical history, alteplase use, endovascular procedures before DC, and modified Rankin scale after 1 year.

### Surgical procedure

Neurosurgeons from our hospital department performed all procedures. Two types of skin flaps were made: a large, inverted question mark (Becker type) and the T-type skin flap. As a department rule, the craniectomy flap was planned to be as big as possible. After craniectomy, the dura was incised, and then an expansion duraplasty using pericranium was made.

### Image analysis

**Figure 1:**
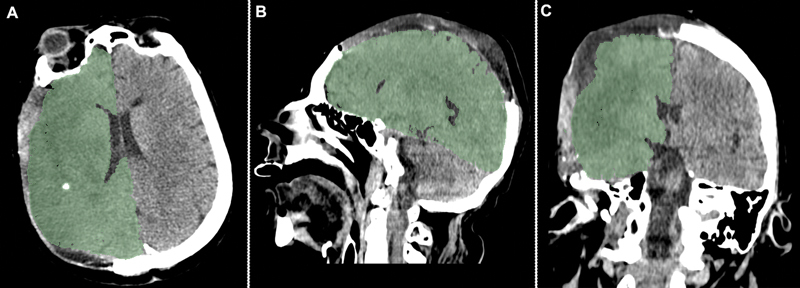
The light green area represents the segmented right hemisphere after decompressive craniectomy, in a postoperative computed tomography exam.

All CT exams performed during these patients' clinical care were analyzed and processed in the software 3D Slicer (v. 4.10 - www.slicer.org ), a free, open-source software, 12 including CT exams before and after DC. After loading the CT scan image on 3D Slicer, we performed whole hemisphere segmentation and calculated the volume of the whole infarcted hemisphere, excluding the ventricular system ( Figure 1 ). To do this, we made the following steps: In *Segment Editor Module* , we started using the threshold of Hounsfield units between 7 and 70 as a mask, to exclude the cerebrospinal fluid (CSF) in the intraventricular and subarachnoid spaces. Then, the whole hemisphere of interest was marked using the automated tool *Grow from Seeds* in the segment editor module. After that, we inspected the quality of segmentation, performing some manual corrections when necessary. Lastly, using the *Segment Statistics Module* , we obtained the volume of the segmented whole hemisphere.

### Statistical analysis

Categorical variables were described as numbers of cases and percentages, and quantitative variables were characterized as mean ± standard deviations (SDs) or means ± range.

In order to demonstrate the average time between the ischemic event and the peak of cerebral hemisphere volume, we used a Kaplan-Meyer survival curve, using the highest measure as the event. If the volumetric variation was seen as a curve (with the highest measure between two or more lower measures), we considered the event's occurrence. The event was censored if the volumetric variation did not show as a curve.

All statistical analyses were performed using SPSS Statistics for Windows, version 17.0 (SPSS Inc., Chicago, IL, USA).

## RESULTS

We identified 53 consecutive patients who underwent a DC after the diagnosis of a malignant MCA infarction in our hospital from 2013 to 2019. Six patients were excluded from the analysis, as we identified hemorrhagic transformation in the ischemic area, and four patients were excluded because of time from symptom onset to DC (higher than 96 hours).

Therefore, we analyzed data from 43 patients (20 female) and a total of 197 corresponding CT exams. The mean age at DC was 52.27 (SD, 11.18] years. The mean time between the ischemic ictus and DC was 41.88 (SD, 29.32) hours ( Table 1 ).

**Table 1:** General characteristics of the patients included in this study

Demographics	
Men	23 (53.5%)
Mean age (years)	52.27 (SD, 11.18]
Mean time from symptom onset to DC (hours)	41.88 (SD, 29.32)
Neurological examination	
NIHSS score 12 (admission)	18 (IQR 17-20)
Ipsilateral mydriasis before DC	12 (27.9%)
ASPECTS 13	
ASPECTS 0	12 (27.9%)
ASPECTS 1	7 (16.3%)
ASPECTS 2	14 (32.6%)
ASPECTS 3-6	10 (23.2%)
Stroke treatment used before DC	
Intravenous alteplase	11 (25.6%)
Intraarterial thrombolysis or thrombectomy	7 (16.3%)
Modified Rankin scale after one year	
mRS 3	16 (50%)
mRS 4	9 (15%)
mRS 5	2 (4.7%)
mRS 6 (death)	16 (37.2%)
Infarction site restricted to ipsilateral MCA territory	25 (58.1%)
Infarction site in MCA territory plus ACA or PCA	18 (41.9%)

Abbreviations: ACA, anterior cerebral artery; ASPECTS, Alberta stroke program early CT score; DC, decompressive craniectomy; IQR, interquartile range; MCA, middle cerebral artery; NIHSS, National Institutes of Health stroke scale; PCA, posterior cerebral artery; SD, standard deviation.

**Figure 2:**
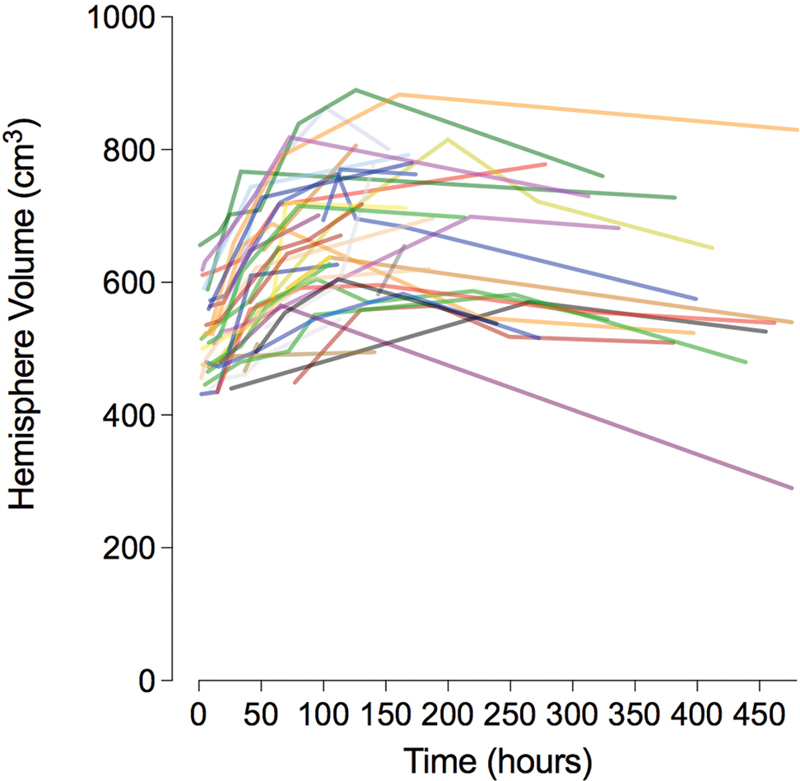
Each colored line represents the course of a single patient.

The time course of the volumetric cerebral hemisphere variation in each patient is shown in Figure 2 .

The mean time between the ischemic event and the peak hemisphere volume was 168.84 (95% confidence interval [CI] [142.08, 195.59]) hours. All patients achieved peak ischemic hemisphere volume after DC.

## DISCUSSION

In this study, we evaluated a group of consecutive patients who underwent a DC after a malignant MCA infarction in our institution. The peak of hemispheric cerebral volume in our patients occurred 168.84 (7.03 days) hours after the initial presentation of malignant stroke symptoms. In all patients, peak hemispheric volume occurred after DC.

Understanding the timing at which ischemic hemisphere volume peaks in these patients is important in managing ICP after DC. Previous studies demonstrated that the ICP could be elevated in patients who underwent DC for malignant cerebral infarction in the postoperative period. 13 14

Dohmen et. al. 3 analyzed a series of 17 patients with MCA infarction and hypoattenuation exceeding 50% of the MCA territory in the early (< 12 h) CT scan. In this series, MLS peaked 62.4 hours after stroke in patients who did not develop malignant edema and 54.7 hours in patients who developed a malignant MCA syndrome. Only two patients underwent a DC in this series.

In our study, all patients underwent DC, and after DC, the MLS became an erratic hemispheric swelling indicator. 15 After DC, many factors could modify the relationship between MLS and hemispheric cerebral swelling, including the area of bone decompression, the adequacy of duraplasty, and the elasticity of the skin. These factors could explain the divergence between the peak of MLS (in previous studies) and the peak of hemispheric cerebral swelling that we identified in our study.

Previous studies have analyzed the volume of infarcted cerebral areas. Brott et al. 16 found a mean volume of infarcted brain tissue (visualized by CT exams performed within the first 48 hours after stroke onset, 7 to 10 days after stroke onset, and 3 months after stroke onset) of 14 cm ^3^ (first 48 hours), 55 cm ^3^ (7-10 days), and 41 cm ^3^ (3 months). In a series of patients with a malignant MCA infarction who underwent DC, Freyschlag et al. 17 observed a mean volume of infarcted brain tissue of 250 cm ^3^ (in the preoperative period), 315 cm ^3^ (postoperative day one), and 349 cm ^3^ (postoperative day three).

In patients with malignant hemispheric cerebral infarction, the whole hemisphere's volume is the main factor that determines the intracranial pressure. Therefore, therapeutic measures have to be based on the time course of the entire hemisphere volumetric variation. Our series demonstrated that the peak volume in the ischemic hemisphere occurred later than previously suspected. Secondary ischemic lesions that occur at the border of the DC area secondary to hemisphere ischemic expansion traction in axonal bundles, among other factors, could explain the delay of the peak hemispheric volume in patients who undergo DC. Furthermore, delayed involvement of PCA or anterior cerebral artery (ACA) territory could also contribute to explain our findings.

As previously expected, the peak ischemic hemisphere volume was after DC in all patients of this series. Preoperative measurements were made to obtain a volumetric baseline for each hemisphere.

Therefore, according to this study's data, after DC in patients with malignant MCA infarction, the critical care team should be vigilant in detecting intracranial hypertension even after the 5 ^th^ day.

The present retrospective study has several limitations. First, it is based on a single institution case series. Second, the hemispheric volumetric calculations were made using a partial manual segmentation method, leading to measurement errors.

In conclusion, the peak of cerebral edema in malignant MCA infarction after DC occurred on the 7 ^th^ day (168.84 h) after stroke symptoms onset. Further studies evaluating therapies for brain edema even after DC should be investigated.
